# Codon Usage Bias in Mitochondrial Genomes Across Three Species of *Siphonaria* (Mollusca: Gastropoda)

**DOI:** 10.3390/genes16070747

**Published:** 2025-06-26

**Authors:** Jingjing Gu, Xuan Zhou, Chao Song, Yiyi Wang, Haobo Jin, Teng Lei, Xin Qi

**Affiliations:** 1 Joint Laboratory of Xianju National Park & Taizhou University, Taizhou University, Taizhou 318000, China; gujj0112@163.com (J.G.); songchaonk@163.com (C.S.); 2 Managing Committee of Xianju National Park, Xianju 317300, China; 3 Zhejiang Key Laboratory for Restoration of Damaged Coastal Ecosystems, School of Life Sciences, Taizhou University, Taizhou 318000, China; zhouxuan030201@163.com; 4 Zhejiang Provincial Key Laboratory of Plant Evolutionary Ecology and Conservation, School of Life Sciences, Taizhou University, Taizhou 318000, China; wangyii4649@163.com (Y.W.); jin01121800@163.com (H.J.)

**Keywords:** mitochondrial genome, *Siphonaria*, codon usage bias, mutation pressure, natural selection

## Abstract

Background: *Siphonaria* is a genus of false limpets belonging to the Gastropoda class. Only two species of this genus have been described with mitochondrial genomes. Moreover, the codon usage patterns and factors influencing them have not been studied. This study aims to expand the mitochondrial genome data of this genus and clarify the codon usage patterns. Methods: The complete mitochondrial genome of *Siphonaria japonica* was sequenced using next-generation sequencing. The gene arrangement and phylogenetic status were compared with *Siphonaria gigas* and *Siphonaria pectinata*. The codon usage bias of the three mitochondrial genomes was analyzed based on the relative synonymous codon usage (RSCU), the effective number of codons (ENC) plot, the parity rule 2 (PR2)-bias plot, and neutrality plot analyses. Results: The gene arrangement and maximum-likelihood phylogenetic tree support a close relationship between *S. japonica* and *S. pectinata*. The codon usage bias analysis indicated that the codon usage bias of mitochondrial PCGs in the three species was primarily influenced by natural selection. Conclusions: This study offers significant evolutionary insights into the phylogenetic relationships and molecular adaptation strategies among *Siphonaria* species.

## 1. Introduction

The false limpets of the genus *Siphonaria* G. B. Sowerby I, 1823 are a group of pulmonates living on intertidal rocks, exhibiting remarkable adaptations to the harsh and dynamic coastal environment. To date, 116 *Siphonaria* species have been described worldwide, showcasing their wide distribution across tropical and temperate marine ecosystems. However, only five species have been recorded in China, i.e., *S. japonica*, *Siphonaria atra*, *Siphonaria sirius*, *Siphonaria petasus*, and *Siphonaria floslamellosa* [1]. Early monographs described *Siphonaria* primarily based on shell morphology, which was considered a key diagnostic feature for species identification. However, significant variations in *Siphonaria* shell morphology have been observed at the species level [2], complicating traditional taxonomic classification. Furthermore, molecular phylogenetic studies have revealed that the characteristics of the reduced shell, often influenced by environmental factors, are insufficient for accurate species delimitation [3]. This highlights the limitations of relying solely on morphological traits in taxonomy. Integrative taxonomy, combining shell morphology and molecular data, could play a more crucial role in the precise delimitation of *Siphonaria* species [4,5].

Maternally inherited mitochondria possess a small genome and exhibit a rapid nucleotide substitution rate, which makes them particularly valuable as genetic markers for evolutionary and taxonomic studies [6]. Mitochondrial DNA (mtDNA) has become a cornerstone in molecular phylogenetics, enabling researchers to trace maternal lineages and assess species divergence with high resolution. Despite their utility, only two species from *Siphonaria* have been described with mitochondrial genomes, i.e., *S. gigas* [7] and *S. pectinata* [8]. These genomes have provided critical insights into the genetic relationships within Gastropoda. Phylogenetic studies on *Siphonaria* species have largely relied on partial sequences of mitochondrial genes such as *COI*, *12S rRNA*, and *16S rRNA*; however, the topology of some nodes was not well supported [1,2,3,4,9]. Comparative genomic analyses incorporating the mitochondrial genome have been widely applied to elucidate evolutionary divergence patterns in aquatic organisms [10]. With the development of high-throughput sequencing technology, the generation of additional *Siphonaria* mitochondrial genomes could significantly enhance the phylogenetic resolution, enabling more robust species delimitation and a deeper understanding of their evolutionary history.

Codon usage bias is a phenomenon wherein some synonymous codons are preferentially utilized over others during protein synthesis [11]. Significant variations in the use of synonymous codons have been observed among different organisms [10,12]. Codon usage bias plays a crucial regulatory role in various cellular processes, such as transcription efficiency, mRNA stability, and translation efficiency [13,14]. For instance, highly expressed genes often favor codons that match the most abundant tRNAs, optimizing the translational speed and reducing errors. The bias is influenced by multiple interacting factors, such as the nucleotide composition, mutation pressure, and natural selection [15,16]. Additionally, environmental stressors such as temperature and nutrient availability can further modulate the codon usage patterns. Therefore, investigating organismal codon usage bias provides valuable data for understanding genome architecture, deciphering species evolutionary relationships, and uncovering molecular mechanisms of environmental adaptation [17].

In this study, we newly sequenced and annotated the complete mitochondrial genome of *S. japonica*, aiming to enrich the mitochondrial data within this genus. Through bioinformatic analyses, we characterized key genomic features including the genome size, nucleotide composition, and the types of start/stop codons across protein-coding genes. Additionally, by integrating mitochondrial genomes from *S. gigas* and *S. pectinata*, the gene arrangement, phylogenetic relationship, and codon usage bias among the three species were analyzed. These multi-dimensional analyses not only expand our understanding of mitochondrial genome evolution in *Siphonaria* but also provide valuable genomic resources for future studies on marine gastropod biodiversity, phylogenetics, and molecular ecology.

## 2. Materials and Methods

### 2.1. Materials Studied

*S. japonica* was collected during low tide from the intertidal zone of Dachen Island, Taizhou City, Zhejiang Province, China (121°54′29.412″ E, 28°27′3.017″ N), in May 2024. Immediately after collection, live specimens were preserved in 75% ethanol and transported to the laboratory under dry ice conditions to maintain DNA integrity. One specimen showing the typical morphological characteristics of *S. japonica* was used for DNA extraction and mitochondrial genome sequencing. The mitochondrial genomes of *S. gigas* and *S. pectinate* were downloaded from GenBank of NCBI under accession numbers NC_016188 and NC_012383. The three mitochondrial genomes were used for phylogenetic and codon usage bias analyses.

### 2.2. Mitochondrial Genome Sequencing

Genomic DNA was extracted from the muscle of *S. japonica* using a DNeasy Blood & Tissue Kit (Qiagen, Hilden, Germany) following the manufacturer’s instructions. The purified DNA was then fragmented by sonication to an optimal size of approximately 250 bp. The fragmented DNA was subsequently sequenced on the DNBSEQ-T7 platform with the PE150 sequencing strategy, generating paired-end reads of 150 bp in length. After sequencing, the raw data were processed to remove low-quality reads and adapters using fastp v0.20.0 [18], resulting in a total of 5.48 Gb of high-quality clean data. To assemble the mitochondrial genome, a partial *COI* sequence (GenBank accession number PQ422951) was utilized as a seed sequence. The NOVOPlasty v4.3.1 assembler [19] was employed, leveraging the seed sequence to generate a contiguous and circular mitochondrial genome assembly. Samtools v1.7 [20] was utilized to calculate the sequencing depth per base, providing insights into the coverage uniformity across the assembled genome. The circular mitochondrial sequence was then annotated using MITOS2 v2.1.9 [21], which identified and annotated all protein-coding genes (PCGs), rRNA genes, and tRNA genes. To gain further insights into the base composition, the GC content and GC skew were calculated using a sliding window of 500 bp. Finally, the mitochondrial genome map, encompassing the genes, GC content, GC skew, and sequencing depth, was visualized using Proksee, available at https://proksee.ca (accessed on 30 April 2025). The complete mitochondrial genome sequence was submitted to the GenBank database of NCBI.

### 2.3. Phylogenetic Analysis

To clarify the phylogenetic status of the three *Siphonaria* species, a maximum-likelihood phylogenetic tree was reconstructed based on a concatenated alignment of mitochondrial PCGs using IQ-TREE v2.4.0 [22]. Prior to tree reconstruction, the 13 PCGs were individually aligned using the MUSCLE method implemented in MEGA X and then concatenated into a single dataset using SequenceMatrix v1.7.8 [23]. The resultant dataset was subsequently submitted to IQ-TREE for phylogenetic analysis. The optimal nucleotide substitution model was automatically selected using Bayesian Information Criterion (BIC). Branch support was assessed through 1000 bootstrap replicates. To root the tree and provide a reference for evolutionary comparisons, *Thuridilla gracilis* (GenBank accession number DQ991939) was selected as the outgroup species, based on its phylogenetic proximity and well-established taxonomic position within the Gastropoda.

### 2.4. Codon Usage Bias Analysis

The relative synonymous codon usage (RSCU), effective number of codons (ENC) plot, parity rule 2 (PR2)-bias plot, and neutrality plot were analyzed to evaluate the codon usage bias of mitochondrial PCGs. The RSCU analysis, which reflects the relative frequency of codon usage when all synonymous codons for an amino acid are equally used [24], was conducted for all PCGs. The RSCU values were calculated using MEGA X [25], with values > 1 indicating preferred codons in the translation process. For the ENC plot analysis, the GC content of the third synonymous position (GC3s) and ENC were calculated using CodonW v1.4.2. The ENC values, ranging theoretically from 20 (extreme bias) to 61 (no bias), were then plotted against the expected curve defined by the formula ENC = 2 + GC3s + 29/[GC3s^2^ + (1 − GC3s)^2^] [26]. Significant deviations below this theoretical curve suggest the presence of selection pressure acting on these mitochondrial genes. The nucleotide composition bias at the third codon position was further examined through PR2-bias plot analysis. A3, T3, C3, and G3 indicate the nucleotide proportions of A, T, C, and G, respectively, at the third codon position in each gene. Their values were calculated using CodonW v1.4.2. In PR2-bias plot maps, G3/(G3 + C3) and A3/(A3 + T3) were set as the x axis and y axis, respectively [27]. The central point (0.5, 0.5) indicates no bias at the third codon position, indicating no significant selection pressure operates at this site. To further distinguish between the mutation and selection pressure in shaping codon usage patterns, we conducted neutrality plot analysis. The GC content of the first, second, third, and all bases of codons (GC1, GC2, GC3, and GCall) for each gene was calculated in Python (v3.9.7). Then, a linear regression of GC12 and GC3 was performed in R, where GC12 represents the average of GC1 and GC2 [28]. A strong linear relationship (slope close to 1) suggests mutation pressure dominates codon usage bias, as GC3 varies proportionally with GC12. To explore potential relationships between different codon usage parameters, Pearson correlation analyses among GC1, GC2, GC3, GCall, and ENC were conducted using the “corrplot” package in R.

## 3. Results

### 3.1. Mitochondrial Genome of S. japonica

**Figure 1:**
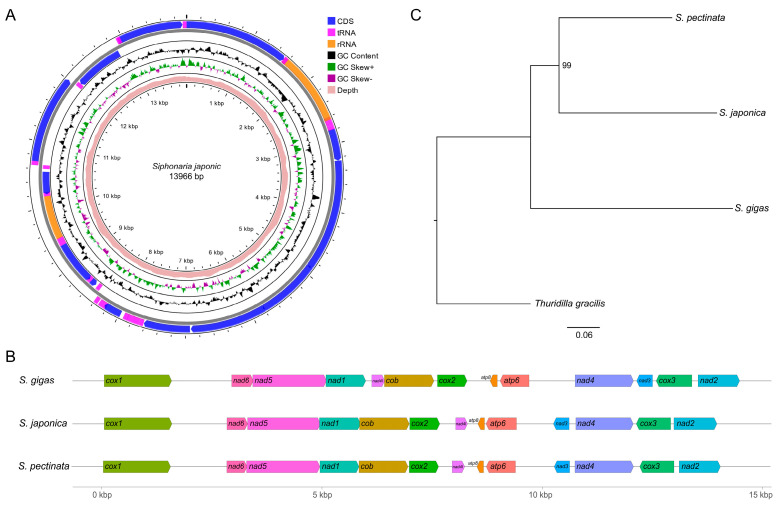
Mitochondrial genome of *S. japonica*. (**A**) Circular map of *S. japonica* mitochondrial genome. (**B**) Gene arrangement of mitochondrial PCGs of *Siphonaria* species. (**C**) Maximum-likelihood phylogenetic tree inferred from *Siphonaria* mitochondrial PCGs. *T. gracilis* was used as the outgroup.

The assembled mitochondrial genome of *S. japonica* was a circular molecule and 13,966 bp in length. Its average sequencing depth was 1848×, and the lowest depth per site was 1319×. The nucleotide composition (28.7% A, 36.8% T, 16.0% C, 18.6% G) presented an A + T bias (65.5%), a negative AT skew (−0.124), and a positive GC skew (0.075). The genome contained 37 genes, comprising 13 PCGs, 22 transfer RNA genes (tRNAs), and two ribosomal RNA genes (rRNAs) (Figure 1A). The PCG region was 10,782 bp and comprised 77.2% of the genome. Nine genes, i.e., *cox1*, *cox2*, *cob*, *nad1*, *nad2*, *nad4*, *nad4l*, *nad5*, and *nad6*, were located on the heavy chain. Four genes, i.e., *cox3*, *atp6*, *atp8*, and *nad3*, were located on the light chain. The gene arrangement was identical to that of *S. pectinata* (Figure 1B). ATG was used as the start codon by *cox2*, *cox3*, *atp6*, *atp8*, *nad2*, and *nad4l*. TTG was used as the start codon by *cox1*, *cob*, *nad1*, and *nad4*. ATT was used as the start codon by *nad3*, *nad5*, and *nad6*. TAG was used as the stop codon by *atp8*, *nad2*, *nad4*, and *nad5*, and other genes used TAA as the stop codon. Among them, the TAA stop codon was completed by the addition of 3′ A residues to the mRNA in *atp6*, *nad1*, *nad3*, and *nad4l*.

### 3.2. Phylogeny of Siphonaria Species

Phylogenetic relationships among three species of *Siphonaria* were reconstructed based on the mitochondrial PCGs. The recovered topology showed two lineages within *Siphonaria*: one included *S. gigas*, and the other included *S. japonica* and *S. pectinata*. *S. gigas* was the most basal ingroup lineage. *S. japonica* was recovered as the sister group of *S. pectinata* (Figure 1C).

### 3.3. Codon Usage Bias of Siphonaria Mitochondrial PCGs

**Figure 2:**
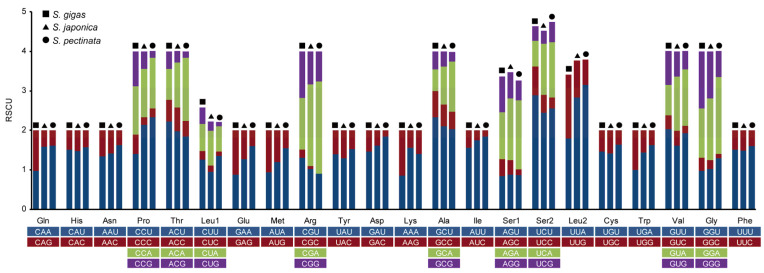
Relative synonymous codon usage (RSCU) of mitochondrial PCGs in *Siphonaria* species.

The RSCU values of all codons corresponding to amino acids in the mitochondrial PCGs from the three *Siphonaria* species were analyzed. The statistical analysis identified 17 high-frequency codons (RSCR > 1) shared among the three species (Figure 2). Of these, 12 ended in U, and 5 ended in A, indicating a bias for high-frequency codons ending in U/A. Notably, nine high-frequency codons were uniquely identified in *S. japonica* and *S. pectinata*, and they all ended in A/U, whereas six high-frequency codons were uniquely identified in *S. gigas*, and they all ended in G. UUA (Leu), UCU (Ser), CGA (Arg), CCU (Pro), and GCU (Ala) were the most frequent codons with the highest RSCU values in both *S. japonica* and *S. pectinata*, and the most frequent codons in *S. gigas* were UCU (Ser), GCU (Ala), ACU (Thr), and GUU (Val).

**Figure 3:**
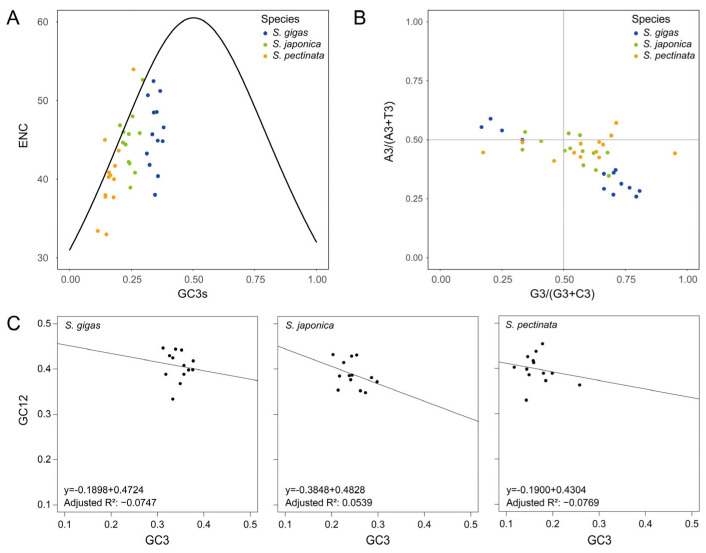
Codon usage bias analyses of mitochondrial PCGs in *Siphonaria* species. (**A**) ENC plot analysis. (**B**) PR2-bias plot analysis. (**C**) Neutrality plot analysis.

The ENC values of mitochondrial PCGs were calculated to investigate the diversity of codon usage bias in the *Siphonaria* species. The mean ENC ± standard error values were as follows: *S. gigas* 45.9 ± 1.2, *S. japonica* 44.8 ± 1.0, *S. pectinata* 40.3 ± 1.5. Because the ENC values of all species were >35, the overall trend of the PCGs showed weak codon usage bias. This weak bias may stem from balanced evolutionary pressures, where translational efficiency, tRNA abundance, and gene expression levels collectively mitigate strong preference for specific codons. Additionally, mutations accumulate more freely in genomes experiencing weak purifying selection. The ENC values were not significantly correlated to the GC content at any codon base in *S. gigas* and *S. japonica*. However, the ENC values were significantly correlated to GC3 in *S. pectinata* (Table 1), indicating that mutational bias at the third codon position contributed to codon preference in this species. The ENC plot showed that all the PCGs of *S. gigas* exhibited ENC values lower than the theoretical values, below the standard curve (Figure 3A), implying that PCGs’ codon usage bias was primarily affected by purifying selection. Purifying selection likely optimizes the translation efficiency via preferred codons matching high-abundance tRNAs. Some PCGs of *S. japonica* and *S. pectinata* were found scattered along the standard curve, and others were below it, indicating diverse codon preference across genes. This suggested that both purifying selection and mutation pressure influenced the PCGs’ codon usage bias. Additionally, factors such as the gene length, protein structure, and epigenetic modifications (e.g., DNA methylation) could further modulate the codon choice.

The impact of mutation and selection pressure on gene codon usage bias was further examined through PR2-bias plot analysis. The analysis revealed that the PCGs of *S. gigas* exhibited a more uneven distribution than those of *S. japonica* and *S. pectinata* (Figure 3B). This asymmetry suggests stronger directional selection in *S. gigas*, potentially driven by adaptation to specific ecological niches or metabolic demands, which preferentially fix G/T-ending codons. The nucleobases G and T were more favored over C and A at the third codon base in most *S. gigas* PCGs. This bias could arise from mutational biases (e.g., oxidative damage favoring G/T) or selection for translational efficiency, as G/T-rich codons may correlate with abundant tRNAs. This trend was also observed in the PCGs of *S. japonica* and *S. pectinata*, while their plots were closer to the center than those of *S. gigas*. The intermediate distribution in these species implies a balance between mutation pressure and weaker purifying selection compared to *S. gigas*. The results suggest that natural selection may be the main factor affecting the codon usage, particularly the third codon base, and *S. gigas* was more influenced than the other two species.

A neutrality plot was used to examine the relationship between GC12 and GC3 to assess the impact of mutation and natural selection on the codon usage bias. The neutrality plot could distinguish between mutational bias (which would show a strong GC12-GC3 correlation) and selective forces (which would disrupt this correlation). The analysis revealed a weak negative correlation between GC12 and GC3, with regression coefficients ranging from −0.3848 to −0.1898 and adjusted R^2^ values ranging from −0.0769 to 0.0539 (Figure 3C). This weak correlation suggests that codon usage bias in *Siphonaria* is primarily shaped by purifying selection rather than mutation pressure, as mutation-driven bias would typically produce a stronger GC12-GC3 relationship. Statistical analysis indicated that there was no significant correlation between GC3 and GC1/GC2 (*p* > 0.05). GC1 and GC2 were both significantly correlated with GCall in all species except *S. japonica*, whose GC2 was not significantly correlated with GCall (Table 1). This observation suggests that while mutational pressures may uniformly influence the GC content at the first and second codon positions, the third position appears to be under distinct selective constraints, likely due to its role in translational efficiency through tRNA abundance. These results indicated that natural selection was the major factor influencing codon usage bias in *Siphonaria*. Selective pressures such as translational efficiency, protein folding kinetics, or ecological adaptations likely drive these biases, overriding neutral mutational effects.

## 4. Discussion

We sequenced the complete mitochondrial genome of *S. japonica*. The genome size was 13,966 bp, slightly smaller than those of *S. gigas* and *S. pectinata*. The PCGs of *S. japonica* and *S. pectinata* presented the same gene order, while they differed from that of *S. gigas*. Gene rearrangements are prevalent across Gastropoda mitochondrial genomes [7]. The same gene order between *S. japonica* and *S. pectinata* indicates their close relationship. The phylogenetic analysis based on mitochondrial PCGs also supported the close relationship between *S. japonica* and *S. pectinata*. The topology of the reconstructed phylogenetic tree among the three species (*S. gigas* + (*S. japonica* + *S. pectinata*)) aligned with that based on the concatenated *COI*, *12S*, and *16S* sequences [4], while it differed from that based on the *16S* sequences [3]. The trees based on one or three makers did not present high bootstrap support values or MCMC posterior probabilities to support their topologies. The continuous supplementation of mitochondrial genome data is expected to provide the molecular foundation for the phylogenetic analysis of *Siphonaria*.

According to the RSCU analysis, *S. japonica* and *S. pectinata* presented similar codon usage bias patterns. This consistency likely reflects shared evolutionary constraints imposed by their tRNA pool composition and translational optimization pressures. The third base of their high-frequency codons was all U/A. Several high-frequency codons of *S. gigas* ended with G. High-expression genes in fast-growing cells particularly benefit from U/A-ending codons by minimizing tRNA competition during rapid protein synthesis. The G-ending preference in *S. gigas* may indicate specialized tRNA modification systems or a unique translational regulation mechanism. The distinct codon ending preference in *S. gigas* also suggests potential evolutionary divergence compared to its counterparts. The preference for high-frequency codons ending in U/A is also observed in plants [29], insects [30], and fungi [31]. This phenomenon suggests that the U/A-ending codon preference might represent an evolutionarily conserved feature across diverse taxonomic groups, potentially linked to translational efficiency or tRNA abundance [32,33]. The divergence in *S. gigas’* codon usage could serve as a molecular marker for studying species-specific adaptation, warranting further investigation through comparative proteomics.

The ENC plot, PR2-bias plot, and neutrality plot analyses all indicated that the codons of the three *Siphonaria* species were predominantly shaped by natural selection, aligning with findings for *Rhingia* [30], *Candida* [31], and *Ganoderma* [34]. The mean ENC value of mitochondrial PCGs varied across different species, implying different codon preferences. This variation may reflect distinct evolutionary trajectories and selective pressures acting on mitochondrial gene expression efficiency. The mitochondrial PCGs of *Siphonaria* species had an average ENC value over 35, demonstrating a weak codon usage bias. Such weak bias suggests relaxed selection or compensatory mechanisms in translational regulation. Higher ENC values were observed in viruses [35], nematodes, flatworms [36], and humans [37]. Lower ENC values were observed in hookworms [38], fungi [31], and nonbiting midges [17]. The ENC plot, PR2-bias plot, and neutrality plot analyses provided evidence that natural selection plays a role in the codon usage bias of *Siphonaria*, which is consistent with the result from the mitochondrial genomes of other species [10,39]. Future comparative studies integrating tRNA gene copy number and expression data could further elucidate the mechanistic basis of these biases.

## 5. Conclusions

We sequenced the complete mitochondrial genome of *S. japonica* and conducted phylogenetic and codon usage bias analyses with *S. gigas* and *S. pectinata*. The results of the gene arrangement, reconstructed phylogenetic tree, and codon usage bias indicated that *S. japonica* had a closer relationship with *S. pectinata*. The codon usage bias analysis also revealed that natural selection predominantly shaped the codon preference of the mitochondrial PCGs of the three species. This study provides crucial evolutionary insights into the phylogenetic relationships and molecular adaptation mechanisms among false limpet species.

## Figures and Tables

**Table 1:** Correlation analysis of codon parameters across mitochondrial PCGs across *Siphonaria*.

(**A**) ***S. gigas***				
	**GC1**	**GC2**	**GC3**	**GCall**
GC2	0.23			
GC3	0.06	−0.30		
GCall	0.87 ***	0.57 *	0.21	
ENC	0.11	−0.46	−0.04	−0.14
(**B**) ***S. japonica***				
	**GC1**	**GC2**	**GC3**	**GCall**
GC2	0.07			
GC3	−0.35	0.01		
GCall	0.73 **	0.56	0.20	
ENC	−0.11	0.39	0.30	0.24
(**C**) ***S. pectinata***				
	**GC1**	**GC2**	**GC3**	**GCall**
GC2	0.38			
GC3	−0.27	−0.02		
GCall	0.75 **	0.69 **	0.32	
ENC	−0.48	−0.27	0.81 ***	−0.03

Significance levels are indicated by * *p* < 0.05, ** *p* < 0.01, and *** *p* < 0.001.

## Data Availability

The data supporting the findings of this study are openly available in GenBank of NCBI (https://www.ncbi.nlm.nih.gov/genbank/, accessed on 20 May 2025). The complete mitochondrial genome of *S. japonica* has been released under accession number PV560261.

## References

[1] Zang G., Wang J., Ma P., Li C., Chen Y., Tang Z., Wang H. (2025). Identifications of common species and descriptions of two new species of *Siphonaria* (Mollusca: Gastropoda) in China. Biology.

[2] Kim Y., Park J., Hwang U.W., Park J.K. (2025). Taxonomic review of Korean *Siphonaria* species (Mollusca, Gastropoda, Siphonariidae). Biodivers. Data J..

[3] Ossenbrügger H., Neiber M.T., Hausdorf B. (2022). Diversity of *Siphonaria* Sowerby I, 1823 (Gastropoda, Siphonariidae) in the Seychelles Bank and beyond. Zool. Scr..

[4] Dayrat B., Goulding T.C., White T.R. (2014). Diversity of Indo-West Pacific *Siphonaria* (Mollusca: Gastropoda: Euthyneura). Zootaxa.

[5] Giribet G., Kawauchi G.Y. (2016). How many species of *Siphonaria pectinata* (Gastropoda: Heterobranchia) are there?. J. Molluscan Stud..

[6] Wang H., Geng S., Liu S., Li Z., Cameron S., Lei T., Xu W., Liu Q., Zuo S., Omongo C.A. (2025). Unraveling the cryptic *Bemisia tabaci* species complex: Global phylogenomic analysis reveals evolutionary relationships and biogeographic patterns. Insect Sci..

[7] White T.R., Conrad M.M., Tseng R., Balayan S., Golding R., de Frias Martins A.M., Dayrat B.A. (2011). Ten new complete mitochondrial genomes of pulmonates (Mollusca: Gastropoda) and their impact on phylogenetic relationships. BMC Ecol. Evol..

[8] Grande C., Templado J., Cervera J.L., Zardoya R. (2004). Phylogenetic relationships among *Opisthobranchia* (Mollusca: Gastropoda) based on mitochondrial *cox 1*, *trnV*, and *rrnL* genes. Mol. Phylogenet. Evol..

[9] Güller M., Zelaya D.G., Ituarte C. (2016). How many *Siphonaria* species (Gastropoda: Euthyneura) live in southern South America?. J. Molluscan Stud..

[10] Lei T., Zheng X., Song C., Jin H., Chen L., Qi X. (2024). Limited variation in codon usage across mitochondrial genomes of non-biting midges (Diptera: Chironomidae). Insects.

[11] Biro J.C. (2008). Does codon bias have an evolutionary origin?. Theor. Biol. Med. Model..

[12] Lei T., Luo N., Song C., Yu J., Zhou Y., Qi X., Liu Y. (2023). Comparative genomics reveals three genetic groups of the whitefly obligate endosymbiont *Candidatus Portiera aleyrodidarum*. Insects.

[13] Liu Y. (2020). A code within the genetic code: Codon usage regulates co-translational protein folding. Cell Commun. Signal..

[14] Quax T.E.F., Claassens N.J., Söll D., van der Oost J. (2015). Codon bias as a means to fine-tune gene expression. Mol. Cell.

[15] Behura S.K., Severson D.W. (2012). Codon usage bias: Causative factors, quantification methods and genome-wide patterns: With emphasis on insect genomes. Biol. Rev..

[16] Das S., Paul S., Dutta C. (2006). Synonymous codon usage in adenoviruses: Influence of mutation, selection and protein hydropathy. Virus Res..

[17] Cao J.K., Lei T., Gu J.J., Song C., Qi X. (2023). Codon bias analysis of the mitochondrial genome reveals natural selection in the nonbiting midge *Microtendipes umbrosus* Freeman, 1955 (Diptera: Chironomidae). Pan-Pac. Entomol..

[18] Chen S. (2023). Ultrafast one-pass FASTQ data preprocessing, quality control, and deduplication using fastp. iMeta.

[19] Dierckxsens N., Mardulyn P., Smits G. (2016). NOVOPlasty: De novo assembly of organelle genomes from whole genome data. Nucleic Acids Res..

[20] Danecek P., Bonfield J.K., Liddle J., Marshall J., Ohan V., Pollard M.O., Whitwham A., Keane T., McCarthy S.A., Davies R.M. (2021). Twelve years of SAMtools and BCFtools. GigaScience.

[21] Donath A., Jühling F., Al-Arab M., Bernhart S.H., Reinhardt F., Stadler P.F., Middendorf M., Bernt M. (2019). Improved annotation of protein-coding genes boundaries in metazoan mitochondrial genomes. Nucleic Acids Res..

[22] Minh B.Q., Schmidt H.A., Chernomor O., Schrempf D., Woodhams M.D., von Haeseler A., Lanfear R. (2020). Corrigendum to: IQ-TREE 2: New models and efficient methods for phylogenetic inference in the genomic era. Mol. Biol. Evol..

[23] Vaidya G., Lohman D.J., Meier R. (2011). SequenceMatrix: Concatenation software for the fast assembly of multi-gene datasets with character set and codon information. Cladistics.

[24] Sharp P.M., Li W.H. (1986). An evolutionary perspective on synonymous codon usage in unicellular organisms. J. Mol. Evol..

[25] Kumar S., Stecher G., Li M., Knyaz C., Tamura K. (2018). MEGA X: Molecular evolutionary genetics analysis across computing platforms. Mol. Biol. Evol..

[26] Wright F. (1990). The ‘effective number of codons’ used in a gene. Gene.

[27] Sueoka N. (1995). Intrastrand parity rules of DNA base composition and usage biases of synonymous codons. J. Mol. Evol..

[28] Sueoka N. (1988). Directional mutation pressure and neutral molecular evolution. Proc. Natl. Acad. Sci. USA.

[29] Ling L., Zhang S., Yang T. (2024). Analysis of codon usage bias in chloroplast genomes of *Dryas octopetala* var. asiatica (Rosaceae). Genes.

[30] Zhao R., Li H., Wu G., Wang Y.F. (2024). Codon usage bias analysis in the mitochondrial genomes of five *Rhingia* Scopoli (Diptera, Syrphidae, Eristalinae) species. Gene.

[31] Wang F., Zhang N., Zhao C., Song Z., Xin C. (2023). Codon usage bias analysis of mitochondrial protein-coding genes in 12 species of *Candida*. J. Genet..

[32] Novoa E.M., Ribas de Pouplana L. (2012). Speeding with control: Codon usage, tRNAs, and ribosomes. Trends Genet..

[33] Drummond A., Shah P., Gilchrist M.A. (2010). Effect of correlated tRNA abundances on translation errors and evolution of codon usage bias. PLoS Genet..

[34] Wu P., Xiao W., Luo Y., Xiong Z., Chen X., He J., Sha A., Gui M., Li Q. (2023). Comprehensive analysis of codon bias in 13 *Ganoderma* mitochondrial genomes. Front. Microbiol..

[35] Shi S.L., Jiang Y.R., Yang R.S., Wang Y., Qin L. (2016). Codon usage in *Alphabaculovirus* and *Betabaculovirus* hosted by the same insect species is weak, selection dominated and exhibits no more similar patterns than expected. Infect. Genet. Evol..

[36] Mazumder G.A., Uddin A., Chakraborty S. (2016). Expression levels and codon usage patterns in nuclear genes of the filarial nematode *Wucheraria bancrofti* and the blood fluke *Schistosoma haematobium*. J. Helminthol..

[37] Chakraborty S., Uddin A., Mazumder T.H., Choudhury M.N., Malakar A.K., Paul P., Halder B., Deka H., Mazumder G.A., Barbhuiya R.A. (2018). Codon usage and expression level of human mitochondrial 13 protein coding genes across six continents. Mitochondrion.

[38] Deb B., Uddin A., Mazumder G.A., Chakraborty S. (2018). Analysis of codon usage pattern of mitochondrial protein-coding genes in different hookworms. Mol. Biochem. Parasitol..

[39] Zhou M., Li X. (2008). Analysis of synonymous codon usage patterns in different plant mitochondrial genomes. Mol. Biol. Rep..

